# Data-Driven Steering Dynamics Modeling and Steering Angle Tracking Control for Self-Driving Vehicles: Simulation and Experiments on 2025 Nissan Leaf Electric Vehicle

**DOI:** 10.3390/s26154827

**Published:** 2026-07-30

**Authors:** Fabrice Simpore, Daniel Vargas, Tadiwa Aubrey Mugwadi, Abdullah Al Tasim, Collin Burch, Jason Ayubu Meshili, Labid Bin Bashar, Yasaman Hajnorouzali, Hanchen Wang, Bin Xu

**Affiliations:** School of Aerospace and Mechanical Engineering, University of Oklahoma, Norman, OK 73019, USAtadiwa.a.mugwadi-1@ou.edu (T.A.M.); abdullah.al.tasim-1@ou.edu (A.A.T.); labid.bashar@ou.edu (L.B.B.); binxu@ou.edu (B.X.)

**Keywords:** self-driving, steering angle tracking, steering dynamics modeling, autonomous vehicles, data-driven modeling and control

## Abstract

Autonomous vehicles (AVs) increasingly rely on accurate steering dynamics models and high-precision steering angle tracking to achieve safe and reliable control. Additionally, the increased attention from automakers and academia emphasizes the potential of AVs in improving transportation safety, convenience, energy efficiency, and ride comfort. This paper presents a data-driven modeling framework using both simulated and real-world driving data collected from a 2025 Nissan Leaf SV Plus. This test vehicle is equipped with an in-house developed drive-by-wire system. Besides the data-driven steering dynamics model, this paper also presents a data-driven steering angle tracking control and a proportional-integral-derivative control. Simulation and on-vehicle tests demonstrate that the proposed data-driven model and controllers can be deployed on the vehicle: the PID controller achieves a steady-state tracking root-mean-square error of 1.23° across the full ±450° operating range, and the neural controller tracks in closed loop with a characterized direction asymmetry identified for future refinement. The proposed data-driven steering dynamics model and control can be used in the development of next-generation autonomous driving systems for their accuracy and simplicity.

## 1. Introduction

As the autonomous driving industry expands, companies such as Waymo, Tesla, Cruise, and other innovators are advancing the deployment of AV technologies for both consumer and commercial applications. These companies emphasize not only the automation of driving tasks but also the enhancement of passenger safety, comfort, and energy efficiency. Effective steering control is a cornerstone of these capabilities, as it directly affects vehicle stability, trajectory tracking, and the ability to safely respond to sudden environmental changes. High-fidelity vehicle dynamics models and real-time control strategies are therefore essential to meet these performance demands [1].

The rapid advancement of autonomous vehicle technologies has emphasized the critical need for precise and responsive steering systems. Traditional steering mechanisms, including hydraulic, electro-hydraulic, and electric power-assisted steering, have evolved considerably over the past decades. However, these systems are still largely constrained by mechanical linkages and offer limited adaptability under dynamic driving conditions. In contrast, drive-by-wire retrofit interface offers a compelling solution for the next generation of AVs, particularly in electric vehicles where efficient integration with onboard electronics is essential [2,3,4]. Modern literature on the subject includes many physics-based and data-driven methods for handling the issue of lane keeping [5,6]. Physics-based models require a much more comprehensive understanding of the vehicle parameters and dynamics that are involved in steering, and thus, development of these models demands more time, knowledge, and capital than data-driven models [7]. To accurately model the physical dynamics experienced by a vehicle in motion, one must consider all the various parameters. Identifying these parameters can quickly become exhaustive, as we consider those related to the “tires, suspensions, powertrain, braking, steering, and aerodynamics,” which are the most prominent areas where such parameters are presented [7]. Additionally, once parameters have been identified, there is no guarantee that they will remain constant throughout the lifetime of the vehicle’s use, as is seen in extensive research on the accurate characterization of vehicle tires in the study conducted by [8].

In this sense, data-driven approaches to steering dynamics modeling have emerged as a particularly powerful tool. By training controllers on large datasets collected from both simulations and real-world driving, these models can capture the inherent nonlinearities of vehicle behavior, adapt to varying road and environmental conditions, and improve steering angle tracking performance. Machine learning models, including decision trees, random forests, and neural networks, allow for the development of controllers that are both computationally efficient and accurate enough for real-time implementation. End-to-end and imitation-learning approaches have demonstrated neural networks mapping sensory or state inputs directly to steering commands [9,10]. Such data-driven methods complement traditional control strategies like PID [11] and model predictive control (MPC), offering enhanced predictive capabilities and adaptability [12]. Recent work has also applied reinforcement learning to coordinate active front steering and direct yaw moment control for vehicle stability under dynamic conditions [13]. Neural-network identification and control of EPS systems for autonomous vehicles has likewise been demonstrated [14], recent surveys chart the rapidly evolving landscape of EPS control strategies [15], and data-driven predictive control has been applied to vehicle stability through digital-twin co-simulation [16]. Broadly, a learned steering controller can be obtained in several ways: by imitation or behavioral cloning of a demonstrated controller [10,17], by end-to-end policy optimization through a differentiable model of the plant using backpropagation through time [18], or by reinforcement learning. End-to-end approaches that optimize a policy against a model are powerful but depend on the fidelity of that model, and controllers learned against imperfect models can fail to transfer to hardware [19]. This motivates the present work choice to develop a comprehensive data-driven steering dynamics model that covers a wide range of operating conditions. Based on this data-driven model, this paper develops both a data-driven steering angle tracking controller and a PID controller and validates both on a 2025 Nissan Leaf SV Plus vehicle.

Modern electric vehicles such as the 2025 Nissan Leaf, employ an electric power steering (EPS) system that retains the conventional mechanical steering linkage between the steering wheel and rack-and-pinion assembly. The proposed system provides electronic steering actuation through the existing EPS architecture, enabling autonomous steering control without permanent modification of the original steering hardware. These vehicles provide an ideal platform to evaluate the effectiveness of the proposed steering controllers under controlled experimental conditions. The combination of advanced control algorithms, high-resolution sensor data, and drive-by-wire retrofit interface hardware enables a comprehensive exploration of steering dynamics, contributing to the broader goals of safety, energy efficiency, and passenger comfort in autonomous driving.

The contribution of this paper can be summarized as follows:

1. Developed an experimental test vehicle platform featuring an in-house developed drive-by-wire system for steering control development based on a 2025 Nissan Leaf vehicle.
2. Developed a data-driven steering dynamics model.
3. Developed data-driven steering angle tracking control.
4. Validated the proposed data-driven steering dynamics model and steering angle tracking control in the 2025 Nissan Leaf test vehicle.

The remaining paper is organized as follows: Section 2 introduces the 2025 Nissan Leaf test platform and experimental data collection setup. Section 3 presents the details of the proposed data-driven steering dynamics model, PID steering angle tracking control, and data-driven steering angle tracking control. Section 4 presents the experimental validation for the proposed controls, followed by Section 5.

## 2. Experimental Setup

**Figure 1:**
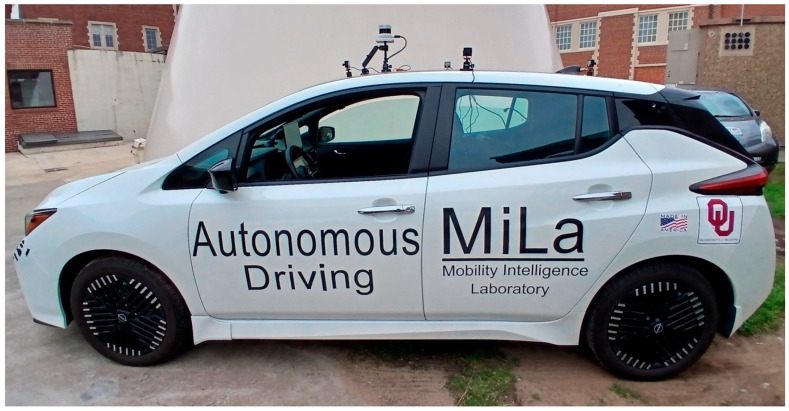
Test vehicle in OU Mobility Intelligence Lab (MiLa): 2025 Nissan Leaf SV Plus electric vehicle.

The data in this study is collected using a 2025 Nissan Leaf SV Plus battery electric vehicle in Norman, Oklahoma, USA as shown in Figure 1 below. It features an Ouster OS1 128 lidar (Ouster Inc., San Francisco, CA, USA), an Xsens MTi-30 IMU (Movella Inc., Enschede, The Netherlands), a Septentrio GNSS (Septentrio N.V., Leuven, Belgium) and four cameras for environmental perception. It is equipped with a 128GB NVIDIA AGX Thor (NVIDIA Corp., Santa Clara, CA, USA) as the on-board companion computer for self-driving tasks. A Comma 3X device (comma.ai, San Diego, CA, USA) is installed for extra data acquisition.

**Figure 2:**
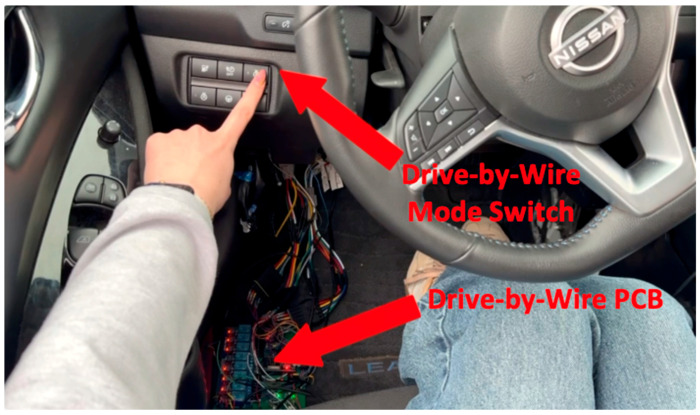
Drive-by-wire retrofit interface steering hardware solution implemented on the 2025 Nissan Leaf test vehicle.

The test vehicle hardware configuration, as shown in Figure 2, consists of our in-house developed drive-by-wire, a Sony DualSense (PlayStation 5, PS5; Sony Interactive Entertainment, Tokyo, Japan) wireless controller for steering input, and an emergency stop button located on the center console. The PS5 controller operates without hardware modification and communicates wirelessly with the primary ESP32 microcontroller (Espressif Systems, Shanghai, China) using the Bluetooth Human Interface Device (HID) protocol. The controller interface is implemented with the open-source Bluepad32 library.

**Figure 3:**
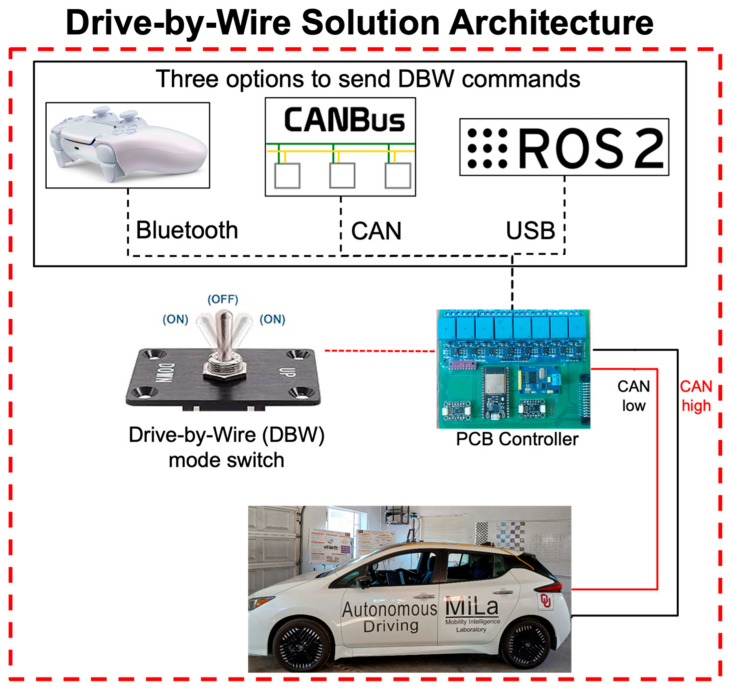
Drive-by-wire solution implemented in the 2025 Nissan Leaf test vehicle.

In this paper, the experimental platform shown in Figure 3 consists of one ESP32 microcontroller, two MCP4728 four-channel digital-to-analog converter (DAC) modules (Microchip Technology Inc., Chandler, AZ, USA), one MCP2515 CAN transceiver module (Microchip Technology Inc., Chandler, AZ, USA), an eight-channel relay board, and an emergency stop switch. Using the PS5 controller, the steering commands are transmitted via Bluetooth to the primary ESP32 microcontroller and to the car. The contribution of this work is the data-driven modeling and control built on this interface rather than the actuation hardware itself. The MCP2515 CAN transceiver is connected to the Comma 3X to acquire steering angle measurements from the vehicle CAN bus. The MCP2515 receives raw CAN frames as hexadecimal payloads. The steering angle and driver torque signals were identified on the CAN bus using SavvyCAN V222: by recording the bus while manually actuating the steering and observing which frame and byte positions varied with the motion, the relevant CAN identifiers and byte layouts were located. The identified signals were then decoded using the appropriate scaling factors and offsets, converting the raw hexadecimal payloads into signed decimal engineering values (degrees for steering angle, and the corresponding units for the driver torque measured by the factory-installed EPS steering torque sensor) before being stored for analysis.

The vehicle was held stationary on rotating supports such that the front wheels could rotate freely without lateral tire forces, decoupling the steering-column dynamics (EPS torque response, mechanical centering, and sensor characteristics) from tire–road interaction. This experimental configuration provides a controlled environment for a clean system-identification dataset of the steering plant actuation and tracking performance, independent of vehicle motion. The data were stored as a CSV file, both on an SD card and via remote connection using a communication node. The remote connection allows for continuous reading of the data for the user, and records the data as a CSV, similar to the SD method. The timestamp, steering angle, and driver torque signals were recorded during a single manual-steering session lasting 6.6 minutes at a 100 Hz sampling rate, yielding 39,726 raw samples. From this recording, the first 25.7 s of static settling were trimmed, leaving 37,153 active training pairs covering steering angles from −468° to +476° and driver torques between −6.7 and +9.7 Nm. The driver intentionally covered a range of steering scenarios to ensure broad coverage of the operating space, as summarized in Table 1.

## 3. Methodology

The research design follows a deliberate progression intended to isolate each source of uncertainty. First, the stationary rotating-support protocol decouples steering-column dynamics from tire–road interaction so that the identified models describe the actuator itself. Second, all five model classes are trained and evaluated under an identical temporal data split, so that performance differences reflect model capacity rather than data handling. Third, the best-performing simple model is used both to tune and to formally verify the PID controller before deployment, and the same step protocol is retained on the vehicle so that simulation and hardware results are directly comparable.

### 3.1. Steering Dynamics Model

#### 3.1.1. Model Introduction

The objective of this section is to identify a discrete-time predictor of the steering actuator that is suitable for controller development rather than to derive a complete physical dynamic model of the electric power steering system. Because the controller operates at 100 Hz, the model predicts the next steering angle using the current steering state, steering torque, and angular velocity. Consequently, the identified model should be interpreted as a local discrete-time steering-state predictor. Once angular velocity is included as an input, the dominant component of the state transition is governed by discrete-time kinematics, while the regression model captures the remaining torque-dependent correction. Throughout this paper, the term “data-driven steering dynamics model” therefore refers to this identified discrete-time input-output predictor rather than a first-principles physical dynamics model. With this requirement in mind, several regression models of increasing complexity are evaluated: linear regression, decision tree, random forest, a compact multilayer perceptron (MLP), and a sequence-based Long Short-Term Memory (LSTM) network.

The linear regression model serves as the baseline. It maps the steering torque and current steering angle to the next steering angle using a relationship. This model assumes that the steering dynamics can be captured by a linear combination of the inputs.

**Figure 4:**
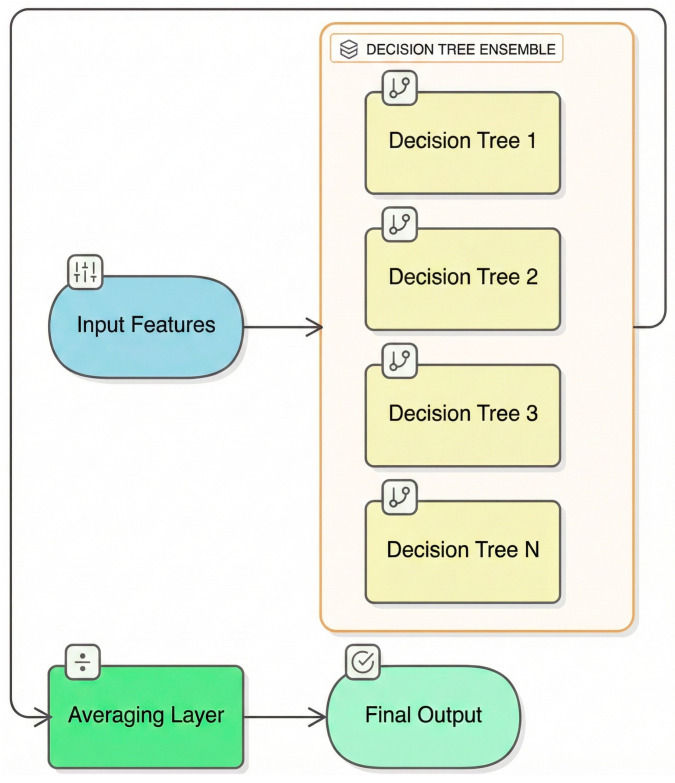
The architecture of the random forest regressor that has been used to estimate the next steering angle from the given inputs.

To account for nonlinearities in the steering dynamics, two tree-based models are then considered: a decision tree regressor and a random forest regressor. The decision tree partitions the input space into regions and fits a simple model (a constant value) within each region, allowing it to capture piecewise-nonlinear behavior. The random forest extends this idea by combining an ensemble of decision trees trained on bootstrapped subsets of the data and features, which typically improves robustness and reduces overfitting. The structure of the random forest used in this study is depicted in Figure 4.

**Figure 5:**
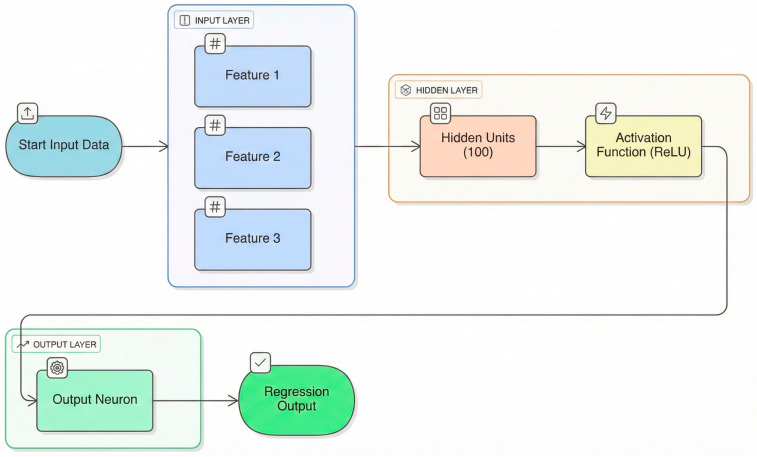
Architecture of the compact multilayer perceptron (MLP) used for next-step steering-angle prediction.

Next, a compact MLP is developed to provide a lightweight, black-box representation of steering dynamics. The MLP was formulated as

(1) $${\hat{\theta }}_{k+1}=f\left(\tau_{1},\tau_{2},\theta_{k}\right)$$

where ${\hat{\theta }}_{k+1}$ is the estimated next steering angle, $\tau_{1}$ and $\tau_{2}$ is the driver torque measured on the steering column via the vehicle CAN bus and $\theta_{k}$ is the current steering angle. The driver torque signal originates from the vehicle’s factory EPS column torque sensor (standard equipment) and is broadcast on the CAN bus; no additional torque instrumentation was installed. To make the second-order steering dynamics observable in a single time step, the angular velocity *ω*_k_ is also supplied as an input feature, computed by finite differences of the angle signal. The network consists of a small number of fully connected layers with nonlinear activation functions, chosen to keep the parameter count low while still capturing nonlinear effects. The architecture of the compact MLP used in this study is shown in Figure 5.

**Figure 6:**
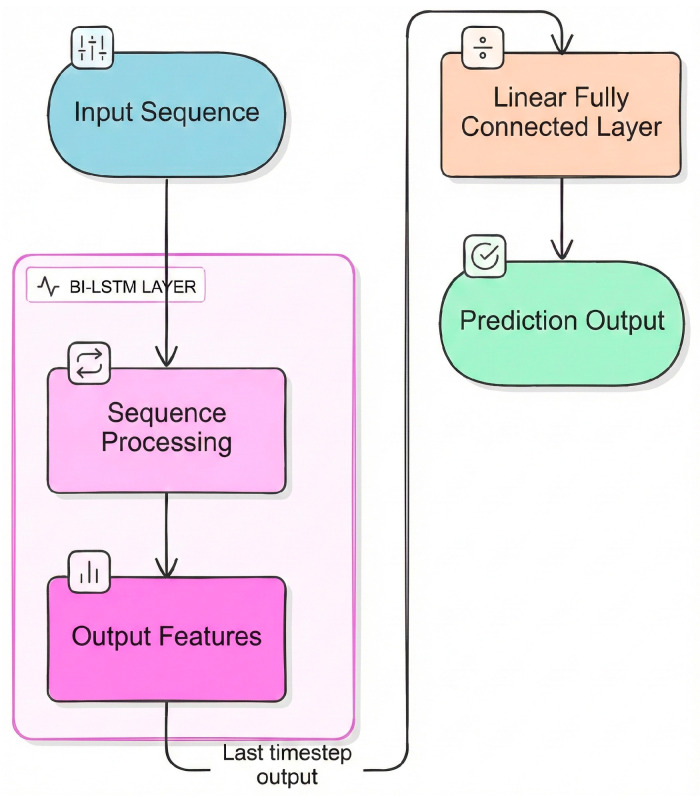
Architecture of the LSTM model used for sequential steering-angle prediction.

Finally, an LSTM network is implemented under the assumption that a recurrent structure might better capture the temporal evolution of the steering dynamics. Instead of predicting the next steering angle from a single time step, the LSTM processes short sequences of steering torque and angles and learns temporal dependencies across multiple samples. In this work, a small LSTM architecture is used to remain compatible with real-time constraints. The structure of the LSTM model is presented in Figure 6.

Together, these five models span a range from simple linear estimators to more expressive nonlinear and sequence-based architectures, allowing a systematic comparison between model complexity, accuracy, and suitability for onboard deployment.

#### 3.1.2. Model Experimental Validation

**Figure 7:**
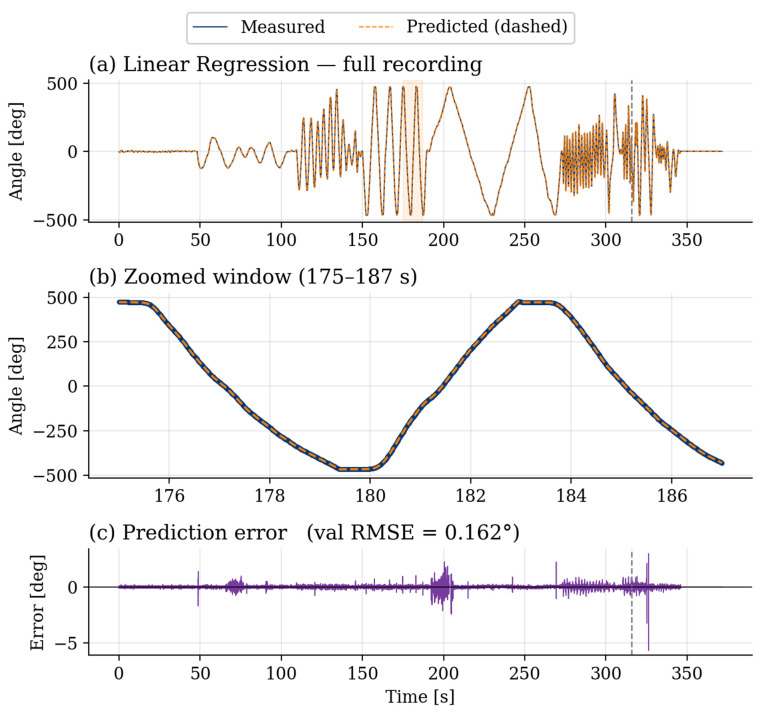
Prediction performance of the linear regression model for next-step steering-angle estimation. The vertical dashed line marks the temporal train/validation split.

All models are trained to predict the next steering angle from the measured steering torque, current steering angle, and angular velocity, using the same dataset and train/test split. After trimming the initial 25.7 s of static settling, the dataset was divided using a time-based (temporal) split: the first 85% of the continuous recording (31,580 samples) was used for training and the final 15% (5573 samples) was held out for validation. A temporal rather than random split was used so that no validation sample is temporally adjacent to a training sample, which would otherwise inflate the apparent one-step accuracy at the 100 Hz sampling rate. For each model, the mean absolute error (MAE) and root mean square error (RMSE) are computed between the predicted and measured next steering angle over all samples in the held-out validation set, and are reported in degrees. The MLP uses two hidden layers of 128 units with ReLU activations, and the LSTM uses a single recurrent layer with 64 hidden units; the random forest uses 100 trees. The linear regression model serves as a transparent baseline. On the comma-collected dataset with angular velocity included as a feature, the linear regression model achieved the lowest held-out validation error of any model considered, with a validation RMSE of 0.162° (MAE 0.066°). However, the high performance of the linear regression model should not be interpreted as evidence that the steering system is fully linear. Rather, it is a consequence of feature engineering inclusion of angular velocity which explicitly accounts for the dominant kinematic evolution of the steering angle, which leaves only a relatively small torque-dependent correction to be identified by the regression model. This can be shown using (*τ*, *θ*, *ω*) representation, the one-step transition is well approximated by $\theta_{k+1}=\theta_{k}+\omega_{k}\Delta t$ plus a small torque-driven correction to account for the previously mentioned inaccuracy, which is itself a linear function of the inputs. This observation explains the low prediction error while also indicating that the identified model represents a discrete-time predictor appropriate for control design instead of a complete physical description of the steering mechanism. Its prediction accuracy relative to the measured steering angle is shown in Figure 7.

**Figure 8:**
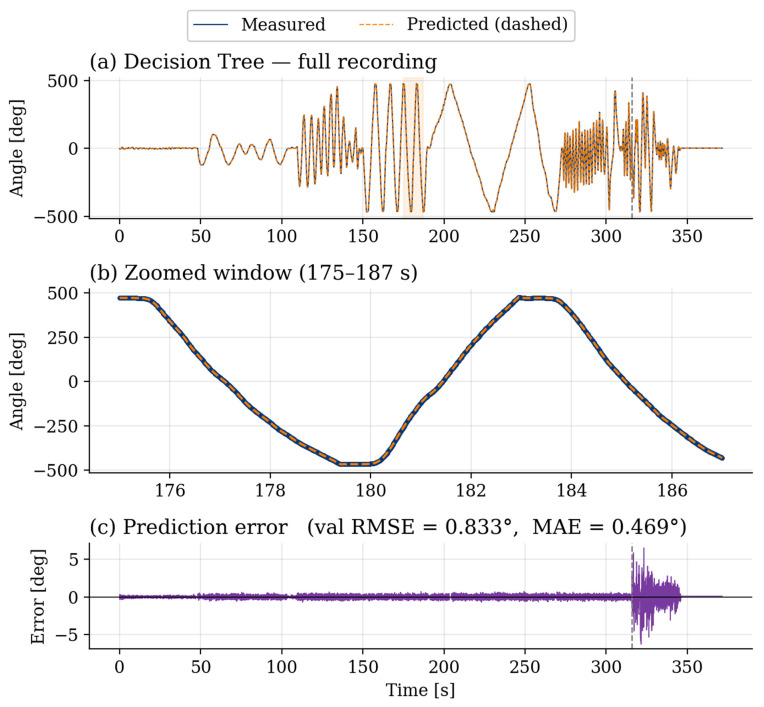
Prediction performance of the decision tree regressor for next-step steering-angle estimation. The random forest results are summarized in Table 2. The vertical dashed line marks the temporal train/validation split.

Two tree-based models are evaluated next to test whether explicit nonlinear partitioning of the input space would improve the linear baseline. Neither did. The decision tree and random forest regressors both produce higher validation error than the linear baseline. As summarized in Table 2, the decision tree achieves a validation RMSE of 0.833° (MAE 0.469°), while the random forest achieves 0.637° (MAE 0.299°). The random forest, which averages an ensemble of trees, generalized better than the single decision tree, but both remain well above the linear baseline. This overfitting is characteristic of axis-aligned tree splits applied to smooth continuous dynamics: the models fit the training trajectory closely but generalize poorly to held-out motion. The decision tree’s predicted steering response compared to the measured dynamics is shown in Figure 8. Despite their nonlinear capacity, the tree-based models do not improve over the linear baseline on held-out data, indicating that the residual nonlinearity in the steering dynamics is small once angular velocity is provided as an input feature.

**Figure 9:**
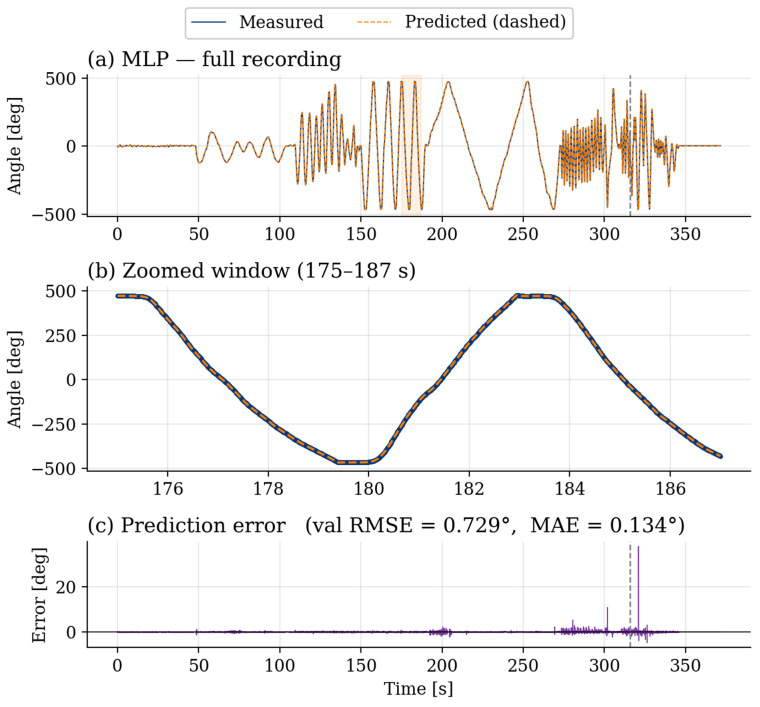
Performance of the MLP model in estimating the next steering angle from steering-torque inputs and current steering state. The vertical dashed line marks the temporal train/validation split.

The compact MLP is retrained on the comma-collected dataset using the driver torque, current angle, and current angular velocity as inputs. On the held-out validation split, the MLP achieves a validation RMSE of 0.729° and a mean absolute error of 0.134°. The gap between these two metrics is informative: the MLP’s MAE is the second lowest of all five models (behind only linear regression), indicating that its prediction tracks the measured angle tightly across the large majority of the recording. Its higher RMSE is driven by a small number of large-error transients in the most aggressive fast-oscillation segments of the validation set, where the squared-error penalty dominates. The MLP is therefore typically very accurate but produces occasional outliers at the hardest operating points. By the universal approximation theorem [20], a sufficiently wide MLP can in principle represent any continuous mapping, so this behavior reflects the difficulty of those specific transients rather than a fundamental capacity limit; the dominant component of the one-step transition remains close to linear in the chosen features, which is why linear regression retains the lowest error. The MLP is nonetheless selected as the forward dynamics model for downstream controller training for three reasons. Neural networks have been used to model vehicle and steering dynamics directly from data, particularly where proprietary power-steering modules make first principles modeling difficult, and such learned models have supported high-performance automated driving [21,22]. First, one-step prediction RMSE is a known weak proxy for closed-loop control performance: when a model is rolled out recurrently inside a controller, small biases or out-of-distribution inputs can accumulate, and the simulation error rather than the prediction error governs deployment quality [23,24]. Second, the MLP’s smooth, fully differentiable mapping integrates naturally with gradient-based controller training, which is the basis of the neural-network controller introduced in Section 3.2.2 and is the standard framework for learned dynamics models used in model-based control [25]. Third, the bounded outputs of a ReLU MLP avoid the unbounded extrapolation of a strict linear fit outside the training distribution, providing more reliable behavior at the operating limits of the steering range. The MLP’s prediction performance is illustrated in Figure 9, where the estimated steering angles closely follow the measured response across the entire recording.

**Figure 10:**
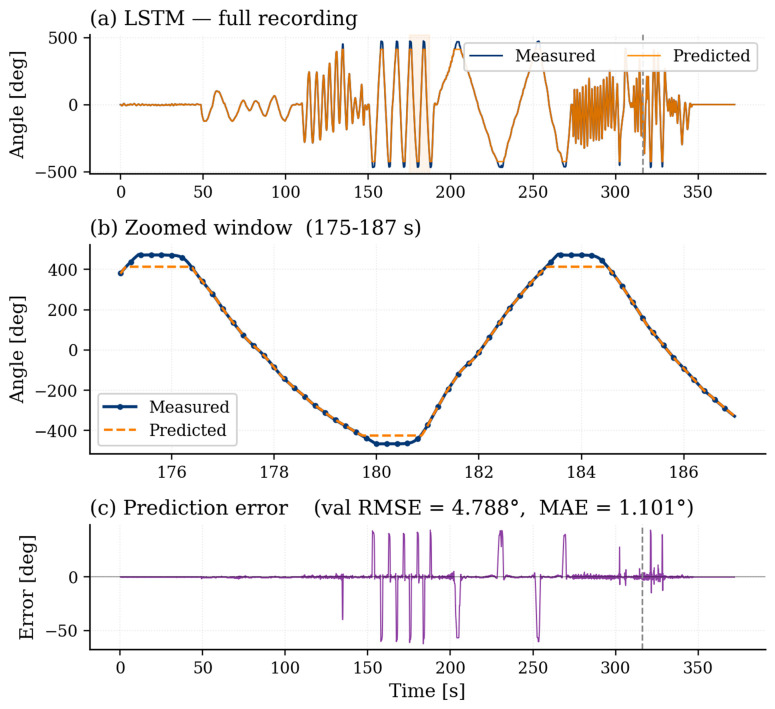
Performance of the LSTM model in estimating the next steering angle. The vertical dashed line in panels (**a**,**c**) marks the temporal train/validation split; the reported metrics are computed on the validation segment to its right.

By contrast, the LSTM network, despite being the most complex model considered, did not improve accuracy over the MLP. Its validation RMSE of 4.788° (MAE 1.101°) is the highest of all five models, more than an order of magnitude above the linear and MLP baselines. The error trace shows that the network tracks small and moderate steering motions accurately but systematically saturates near the limits of the steering range: at the full-lock plateaus it clips to roughly ±410° and fails to reach the measured ±470° extremes, producing large errors precisely at the most demanding operating points. This underfitting of the range extremes, together with the lack of improvement on the bulk of the trajectory, indicates that once angular velocity is supplied as an explicit feature the temporal context the recurrent structure is intended to capture is already present in the single-step input. The added training and recurrent inference cost therefore do not pay off for this task. The estimation performance is presented in Figure 10.

**Figure 11:**
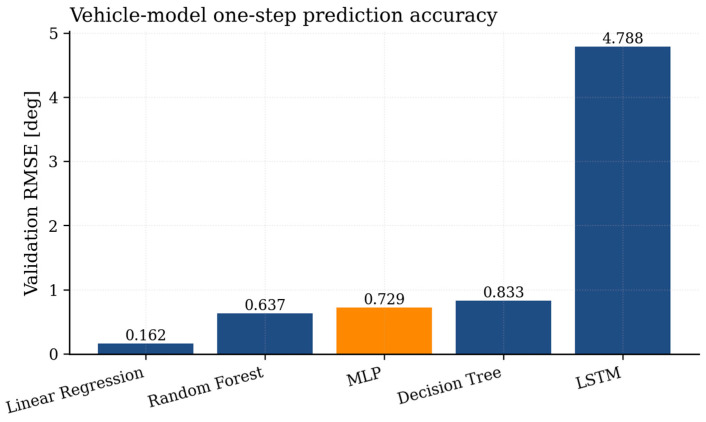
Validation RMSE for the five steering dynamics models. The MLP (highlighted) is carried forward to the controller-design stage despite not achieving the lowest RMSE.

Overall, the results in Table 2 and Figure 11 show that one-step prediction accuracy alone is not sufficient for controller selection. The linear regression model achieved the lowest one-step RMSE and MAE, and was therefore selected as the simulation model for PID controller development because of its numerical stability, transparency, and predictable behavior during recursive closed-loop simulation. In contrast, the MLP was selected for the neural controller because the proposed controller requires a differentiable forward model from which an inverse mapping can be learned using gradient-based optimization [25]. The tree-based models were not considered for controller development because their piecewise-constant structure is not differentiable and therefore unsuitable for inverse-model learning, while the LSTM exhibited substantially poorer prediction accuracy than the other candidates. The motivation is twofold:(1) identifying the most accurate and numerically reliable forward model for classical control development and (2) selecting an appropriate differentiable nonlinear model for data-driven controller design. The subsequent controller comparison therefore evaluates not only different control strategies but also the suitability of different forward-model classes for steering control. One-step RMSE is not equivalent to closed-loop control performance: in recurrent rollouts inside a controller, the relevant quantity is the simulation error, which can differ markedly from the prediction error for nonlinear or biased models [23,24]. Because the comparison between linear and nonlinear simulators is itself relevant to the practical question of which forward model leads to better controllers, both candidates are carried through to the next section, where their corresponding PID and neural-network controllers are evaluated in closed loop.

### 3.2. Steering Angle Tracking Control

#### 3.2.1. PID Steering Angle Tracking Control

The development of a forward steering dynamics model enables the design and tuning of a classical PID controller as a baseline control of this paper which is evaluated in closed-loop simulation. The linear regression model is selected as the closed-loop simulator for this stage: because its dynamics are globally smooth and bounded in the operating region, it remains well-posed when driven by controller-generated inputs, whereas the MLP, despite its strong one-step accuracy, can become unstable outside the distribution of driver torques it was trained on. This is consistent with the established distinction between prediction error and simulation error in learned dynamics models [23,24], and the MLP is instead reserved as the differentiable simulator for the neural-network controller in Section 3.2.2. The PID controller computes a control input $u_{k}$ based on the reference steering trajectory $\theta_{ref}$ and the current steering angle $\theta_{k}$. Since the physical steering actuators operate within a limited range, the control input is saturated, i.e., $u_{k}\in [-390,\;390]$. $u_{k}$ is then mapped to the left and right steering actuators as:

(2) $$u_{1,k}=2469+u_{k},$$

(3) $$u_{2,k}=2469-u_{k}$$

These values, together with the current steering angle and angular velocity, are fed to the linear forward steering model to predict the next steering angle:

(4) $$\theta_{k+1}=f_{1}\left(u_{1,k},u_{2,k},\theta_{k}\right)$$

**Figure 12:**
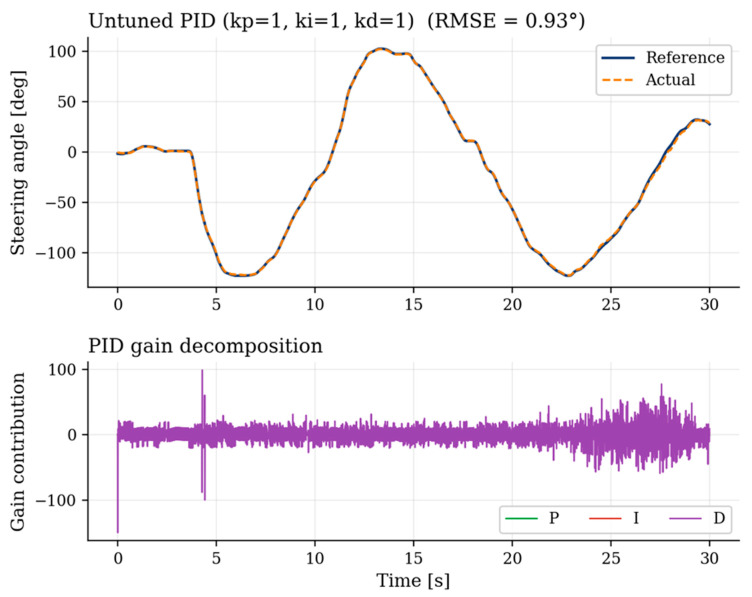
(**Top**) Closed-loop PID steering response (RMSE=0.93°). (**Bottom**) Decomposition of PID gain contributions for the untuned controller parameters. The integral and derivative contributions remain near zero at this scale and are visually covered by the dominant proportional term; all three legend entries are plotted.

This produces a simulated PID tracking response tracking the reference shown in Figure 12. The tuning process is done manually, starting off with $k_{p}=1,\;k_{d}=1,\;k_{i}=1$.

**Figure 13:**
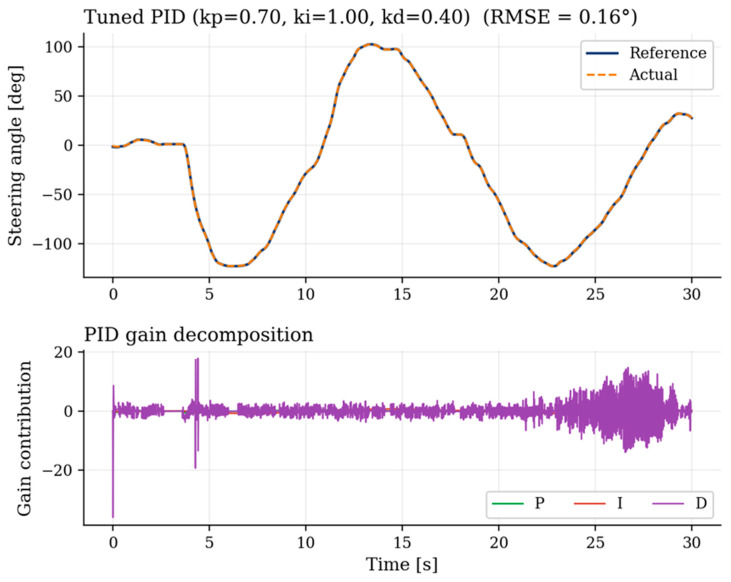
(**Top**) Closed-loop PID shows the steering response (RMSE = 0.16°). (**Bottom**) Decomposition of PID gain contributions for $k_{p}=0.70,\;k_{i}=1.0,\;and\;k_{d}=0.40$.

From the gain decomposition of the untuned controller, the derivative term exhibits noisy, high-frequency action with transient spikes reaching roughly ±100. With unity gains, the controller tracked the reference only loosely, and the control effort was dominated by this noisy derivative response. Adding stronger integral action to eliminate steady-state offset and a larger, low-pass-filtered derivative term to damp the response substantially improved tracking. This corresponds to increasing both $k_{d}$ and $k_{i}$. The proportional gain $k_{p}$ is held at a moderate value to avoid overshooting and keep the loop stable. The final controller configuration has $k_{p}=0.70,\;k_{i}=1.0$ and $k_{d}=0.40$ which produces the results shown in Figure 13.

**Figure 14:**
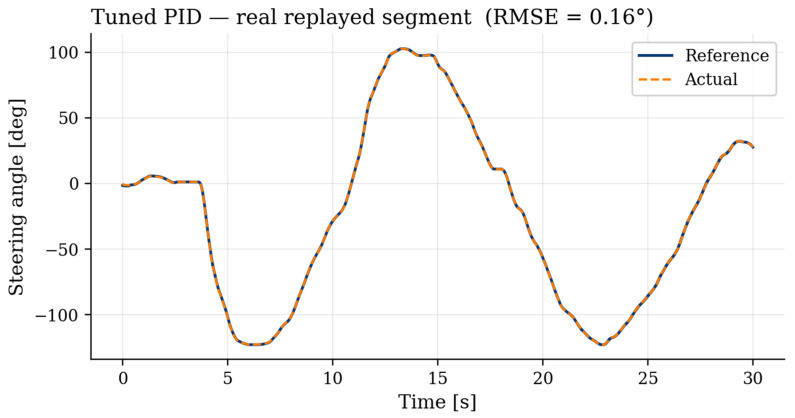
Tuned PID tracking a real recorded steering segment (RMSE 0.16°, MAE 0.12°).

**Figure 15:**
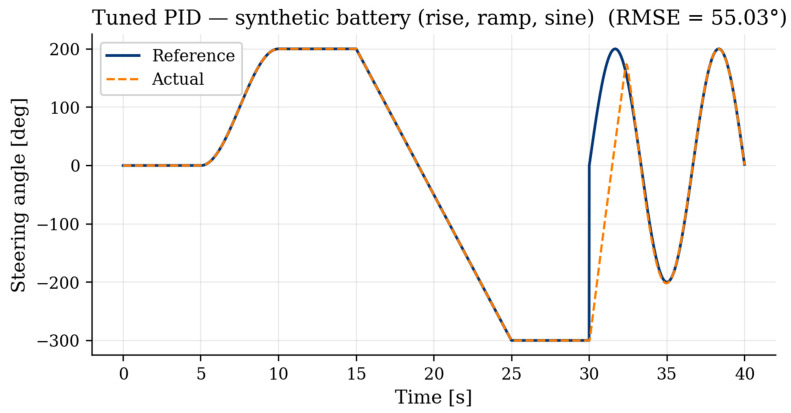
Tuned PID on a synthetic stress reference (smooth rise, full-range ramp, and continuous sine).

To assess the tuned controller on realistic inputs, the closed loop is driven by a reference taken from a recorded segment of manual driving (moderate turns reaching roughly 120° at angular rates near 25°/s). On this real reference the tuned controller tracks the target with an RMSE of 0.16° and a mean absolute error of 0.12°, as shown in Figure 14. A synthetic stress reference combining a smooth rise, a full-range ramp, and a continuous sine sweep is also evaluated (Figure 15); tracking remained accurate through the ramp and low-frequency sine, with the residual error concentrated at the sharpest transitions, where the rate-limited steering plant cannot instantaneously follow the commanded change.

#### 3.2.2. Data-Driven Control

Once a suitable PID controller has been built, a neural network data-driven controller is investigated as an alternative to the PID.

This data-driven approach involves 3 stages: (I) synthetic data generation using the feedforward steering model, (II) training a neural network to approximate the inverse steering mapping, and (III) closed-loop evaluation in which the learned inverse model is applied as a feedforward term together with integral feedback.

(I) Data Generation Using the Forward Steering Model

Rather than training the inverse controller directly from the experimental dataset, the forward model is first used to generate a dataset of 10,000 data points for synthetic excitation data. The data is generated by repeatedly applying steering trajectories control inputs, $u_{k}\in [-390,390]$, to the trained MLP Regressor feedforward steering dynamics model. The input distribution is similar to that with a real driver’s behavior and does not uniformly excite the input space required for inverse-model identification. This data approach follows the traditional model-based learning controller where a forward-model is first identified then inverted through supervised learning. The dataset is stored as:

(5) $$D={\left\{\theta_{k},u_{k},\theta_{k+1}\right\}}_{k=1}^{10,000}$$

(II) Approximate a data-driven steering controller

The goal of the controller is to produce $u_{k}$ required to move from the current steering angle $\theta_{k}$ to the desired next value $\theta_{k+1}$. This corresponds to learning the inverse mapping:

(6) $$u_{k}=f_{2}\left(\theta_{k},\;\theta_{k+1}\right)\;$$

To achieve this, an MLPRegressor is trained using the dataset generated in (I). The MLPRegressor is evaluated on a 70/30 testing and training split. The inverse controller only achieves an $R^{2}=\;0.88$ indicating that the inverse mapping is well learned from synthetic data. Any approximation error present in the forward model is propagated to the inverse model. For this reason, the learned inverse controller is not deployed as a stand-alone controller but is combined with integral feedback that compensates residual modeling error, steady-state bias, and plant uncertainty. Direct training of an inverse controller using only the available experimental data is left for future investigation.

(III) Closed-Loop Evaluation

**Figure 16:**
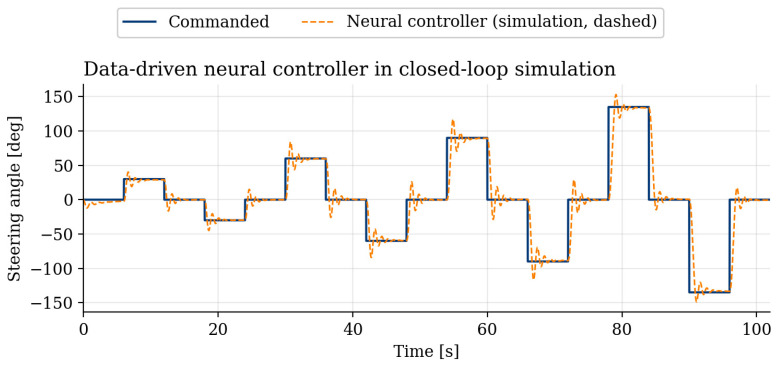
Data-driven neural controller (learned inverse-model feedforward with integral feedback) in closed-loop simulation against the vehicle-identified velocity-lag plant, tracking at approximately one-degree RMSE. The controller tracks the step sequence symmetrically in both directions; transient overshoot reflects the oscillatory identified plant.

To evaluate the controller’s performance, a closed-loop simulation is run against the velocity-lag plant identified from the vehicle: the angular velocity follows *ω*(*k* + 1) = A·*ω*(*k*) + B·*u*(*k*) + C·*θ*(*k*) + D with the angle integrated as *θ*(*k* + 1) = *θ*(*k*) + *ω*(*k* + 1)·Δ*t* at Δ*t* = 0.02 s, where u is the commanded steering torque in the actuation units of the drive-by-wire interface. The coefficients identified by least squares from recorded on-vehicle closed-loop data are A = 0.969, B = 0.071, C = −0.030, and D = −6.06 (R^2^ = 0.96); unlike the one-step predictors of Section 3.1, this plant captures the actuator lag that dominates closed-loop behavior. Because a one-step inverse model applied alone is ill-posed for a plant with significant actuator lag and is sensitive to the plant’s steady-state bias, the learned inverse network is used as a feedforward term and combined with integral feedback on the tracking error. The feedforward term, the learned inverse mapping $f_{2}(\theta_{k},\;\theta_{k+1})$, provides an approximate control to move toward the commanded setpoint, while the integral term accumulates the residual tracking error and cancels the steady-state bias of the identified plant. A smoothed setpoint and a rate limit on the commanded torque keep the loop within the responsive region. Figure 16 shows the resulting controller in closed-loop simulation against the velocity-lag plant. The controller tracks the step sequence in both directions across the full amplitude range with a steady-state RMSE of approximately one degree, with transient overshoot at the larger amplitudes that reflects the oscillatory character of the identified plant. The tracking is symmetric in the two directions. We note that the integral feedback is responsible for driving the steady-state error to zero; the learned inverse term contributes an approximate feedforward command that reduces the burden on the feedback loop but is not by itself sufficient for stable closed-loop tracking.

## 4. Experimental Validation

### 4.1. PID Control Deployment

The tuned PID controller is deployed on the Nissan Leaf to validate the simulation-based design on real hardware. The controller runs on an ESP32 that reads the steering angle from the vehicle CAN bus (frame 0 × 2, decoded as a little-endian signed integer scaled to degrees) and commands steering torque by injecting analog signals into the electric power steering torque-sensor lines through dual MCP4728 digital-to-analog converters. The control loop, setpoint smoothing, saturation, and slew limiting mirror the simulated controller. The gains are re-tuned on the vehicle from the simulation values to account for the difference between the identified model and the real steering system, yielding kp = 1.2, ki = 1.5, and kd = 0.3 on hardware; this on-vehicle tuning is expected, since the simulator captures the dominant dynamics but not every effect of the physical electric power steering system. Because the identified linear model provides an explicit discrete-time plant, the closed-loop stability of these gains can be verified directly: with the state augmented by the integral of the tracking error, all closed-loop poles lie inside the unit circle for the selected gains, and a sweep of the integral gain shows the loop remains stable for k_i up to approximately 2.5, so the chosen k_i = 1.5 lies within the stable region with margin. A formal stability guarantee for the nonlinear data-driven controller is left to future work. To exercise the full operating range observed in the recorded driving data, the controller is commanded through a repeatable step sequence spanning small to large amplitudes (15, 30, 60, 90, 135, 180, 270, 360, and 450 degrees in both directions), returning to center between each step and holding each setpoint long enough to reach steady state. Every sample is streamed over Wi-Fi to a host computer for analysis.

**Figure 17:**
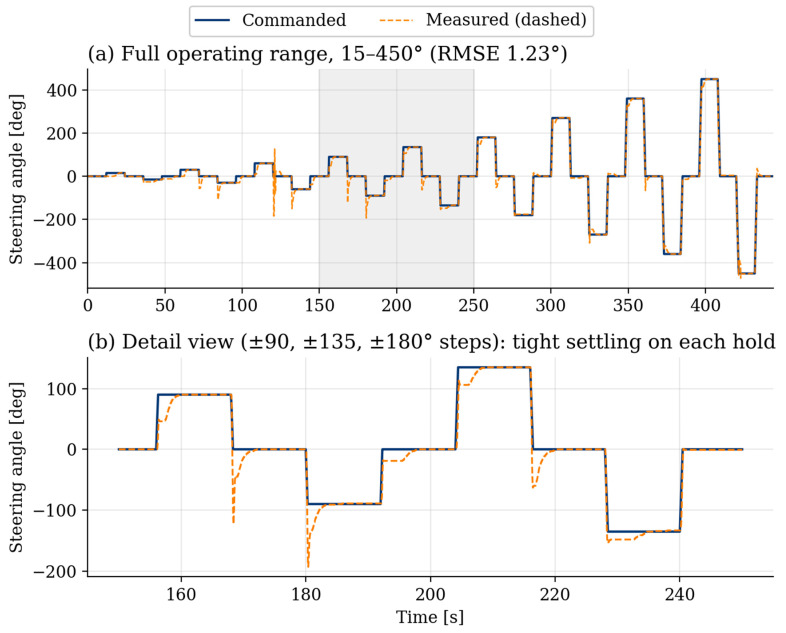
On-car PID steering tracking. (**a**) Full operating range, 15–450° in both directions (overall steady-state RMSE 1.23°); the shaded region is expanded in (**b**). (**b**) Detail view of the ±90, ±135, and ±180° steps, showing a brief transient at each step edge followed by tight settling on the hold (RMSE 0.69° in this window).

**Figure 18:**
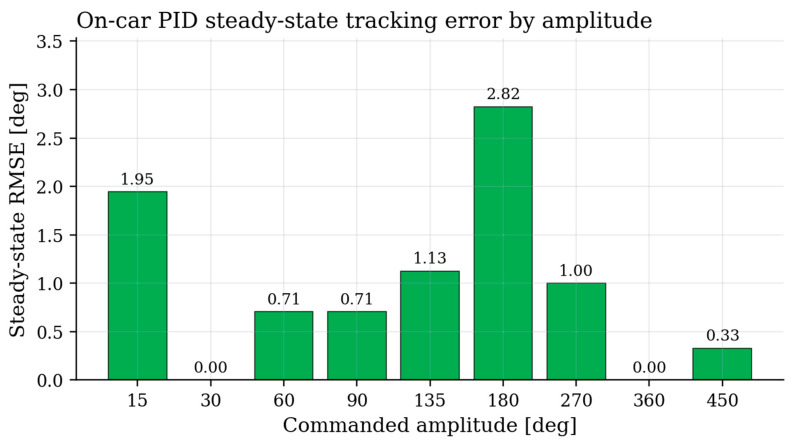
On-car steady-state tracking error by commanded amplitude. Error stays within ~3° across the full range, most amplitudes within ~1°.

Figure 17a shows the measured steering angle tracking the commanded reference across the full sequence, and Figure 17b expands a representative window. The controller follows each step closely; the larger steps exhibit a brief transient at the step edge before settling tightly on the target, with steady-state error in the expanded window of 0.69 degrees. Computing the steady-state error over the settled portion of each hold yields an overall root-mean-square tracking error of 1.23 degrees across the entire range. Figure 18 breaks this down by commanded amplitude: the steady-state error remains small and roughly flat from the smallest to the largest commands, with all amplitudes tracked to within approximately 3 degrees and most within about 1 degree. Notably, the large-amplitude commands up to 450 degrees reach their targets without the steady-state droop seen with weaker integral action, confirming that the controller maintains accurate tracking across the vehicle’s full steering range with no electric-power-steering fault conditions triggered during testing.

### 4.2. Neural-Network Control Deployment

**Figure 19:**
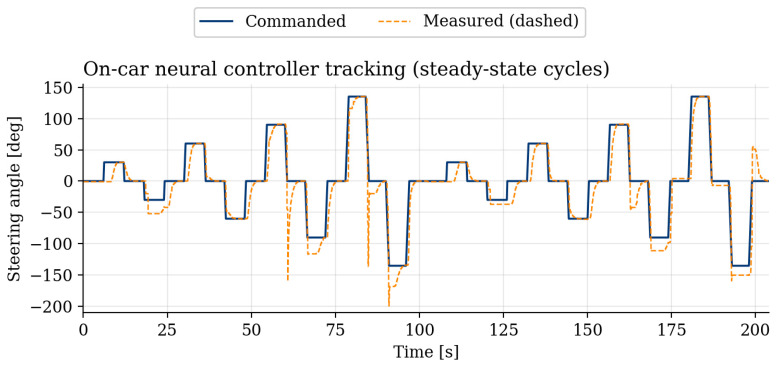
On-car neural controller tracking over the steady-state cycles of the validation sequence. The controller follows commanded steps in both directions; isolated spikes are residual CAN feedback glitches.

**Figure 20:**
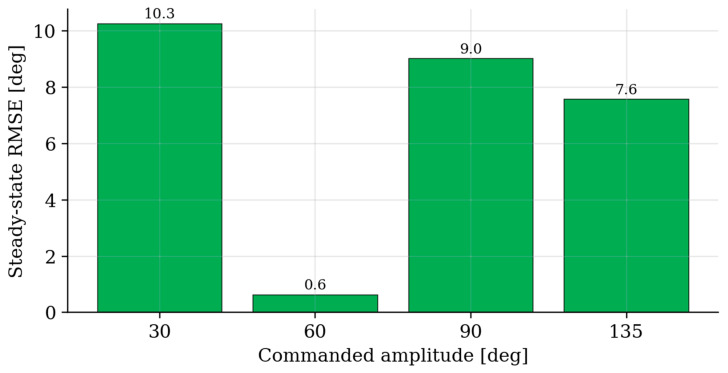
On-car steady-state tracking error by commanded amplitude (steady-state cycles, both directions combined).

The neural network control is deployed using the same firmware envelope and step sequence as the PID. The firmware maintains the integral term used by the feedback loop and evaluates the learned network at each control step. To reject occasional corrupt CAN angle frames, a non-latching outlier filter was added that discards a single physically impossible jump but accepts sustained motion, so that legitimate fast reversals are not rejected. The neural controller runs the full three-cycle validation sequence in a closed-loop on the vehicle. In the steady-state cycles it tracks the commanded steps in both directions (Figure 19), reaching the positive-direction targets within about one degree. Figure 20 summarizes the steady-state error by amplitude.

Two limitations are noted. First, the neural controller exhibits a direction asymmetry on the vehicle: positive-direction commands settle within about one degree, while negative-direction commands overshoot the target by roughly eight to fifteen degrees. The neural-controller vehicle tests reported here used the same step protocol family as the PID tests (identical hold times, target smoothing, slew limiting, and CAN feedback path) but covered commands only up to ±135°, whereas the PID was validated across the full 15°–450° range; the quantitative PID-versus-neural comparison is therefore restricted to the amplitude range covered by both controllers, and completing full-range neural-controller validation under the identical protocol is an immediate next step. This asymmetry does not appear in the closed-loop simulation, which is symmetric, and it does not appear in the PID hardware data on the same vehicle. Its presence with the neural controller but not the PID indicates that it is not a pure hardware bias but an interaction: the network approximates the control mapping but not the full filtered-derivative and timing behavior of the feedback loop, and this approximation gap interacts with a directional characteristic of the electric power steering to produce the overshoot. Second, the controller remains sensitive to corrupt CAN feedback. While the outlier filter prevented the catastrophic feedback freeze seen in an earlier deployment, a burst of glitches during the first cycle still produced a transient excursion before the controller recovered and completed the remaining cycles cleanly; the steady-state results above are reported from those clean cycles. Both limitations point to clear future work: refining the neural controller to remove the direction asymmetry and improving the integrity of the CAN angle feedback. The central result stands: a neural controller runs in closed loop on the vehicle and tracks the commanded steering across the operating range.

## 5. Conclusions

This work develops and validates a steering dynamics model and a steering angle tracking control for a 2025 Nissan Leaf self-driving test platform, progressing from data-driven dynamics models through controller design to on-vehicle deployment. Five steering-dynamics models are identified from recorded driving data at a 100Hz sampling rate, and the resulting simulator is used to design and tune a PID controller. Deployed on the vehicle, the PID tracks commanded steering across the full operating range from 15 to 450 degrees in both directions, with an overall steady-state root-mean-square error of 1.23 degrees and no electric-power-steering faults; the controller gains required modest re-tuning on the vehicle relative to their simulated values, reflecting the gap between the identified model and the physical steering system. Table 3 consolidates the tracking performance of both controllers across simulation and vehicle testing.

A data-driven controller is then obtained. Implemented as a compact rectified-linear network, the neural controller runs in closed loop on the vehicle and tracks the commanded steering across the operating range. Its limitations are characterized: a direction-dependent overshoot that appears on the vehicle but not in simulation, indicating an interaction between the approximation and a directional steering characteristic, and a residual sensitivity to corrupt CAN feedback that a non-latching outlier filter mitigates but does not eliminate.

Taken together, the results show that a classical controller, carefully identified and tuned, provides a robust and accurate baseline for automated steering, and that a learned neural controller can be deployed on the vehicle to track steering across the operating range. Future work includes refining the neural controller to remove the direction asymmetry, improving the integrity of the CAN angle feedback, and extending the identified plant with designed excitation experiments so that learned controllers can be trained and verified against dynamics that capture the actuator lag of the physical steering system. Comparisons against first-principles vehicle models and against advanced controllers such as MPC and sliding-mode control, under in-motion conditions, and an end-to-end controller trained by backpropagation through a differentiable plant are left to future work.

In the future, more comprehensive experimental study is planned to be conducted. In addition, path following algorithms will be developed for the point-to-point autonomous navigation task, which is extremely important in self-driving research. The immediate next step is closed-loop path tracking on the same platform, with the characterized steering actuator serving as the inner loop of a hierarchical controller, so that the actuator nonlinearities identified here can be explicitly compensated at the path-tracking level.

## Figures and Tables

**Table 1:** Manual steering scenarios captured during the 6.6 min data collection session. Each scenario covers a distinct class of steering motion to ensure broad coverage of the operating space.

Scenario	Description	Steering Range
Center driving	Small corrections around straight	Within a quarter turn
Gentle turns	Smooth turns to moderate angles	Up to about half a turn
Sharp turns	Rapid turns to large angles	Up to about one full turn
Full-lock maneuvers	Steering driven to mechanical stops	Full lock either side
Slow continuous sweeps	Smooth lock-to-lock motion	Full operating range
Fast oscillations	Rapid back-and-forth steering	Moderate amplitude
Freestyle driving	Mixed natural steering motion	Varied

**Table 2:** One-step validation of RMSE and MAE for the five steering-dynamics models, ordered by RMSE. Linear regression achieves the lowest error on both metrics.

Model Name	Training Complexity	RMSE (deg)	MAE (deg)
Linear Regression	Fast/Low	0.162	0.066
Random Forest	Fast/Low	0.637	0.299
MLP	Fast/Moderate	0.729	0.134
Decision Tree	Fast/Low	0.833	0.469
LSTM	Slow/High	4.788	1.101

**Table 3:** Summary of steering-tracking performance for both controllers in simulation and on the vehicle.

Controller	Stage	Test Range	Tracking Result
PID	Simulation	Step sequence, full range	0.16° tracking RMSE
Neural network	Simulation	Step sequence, full range	≈1° steady-state RMSE
PID	Vehicle	15–450°, both directions	1.23° steady-state RMSE; no EPS faults
Neural network	Vehicle	Up to ±135°	≈1° (positive); 8–15° overshoot (negative)

## Data Availability

The data presented in this study are available on request from the corresponding author.
